# Sir J. Sinclair on Health and Longevity

**Published:** 1805-06-01

**Authors:** John Sinclair

**Affiliations:** 5, Terrace, Palace Yard, Westminster


### *To the Editors of the Medical and Physical Journal.*

GENTLEMEN,

IN the course of the very extensive Inquiries regarding health and longevity, in which I am at present engaged, it naturally occurred, that much useful information might be obtained, from ascertaining the means by which persons who are trained to athletic exercises are brought to that height of strength and activity which it is necessary for them to acquire; and by what processes the speed, the strength, and the courage, of the brute creation can be improved. With a view of obtaining a knowledge of the facts from which such important consequences result, the enclosed paper, and the queries therein contained, were drawn up.

As the insertion of that paper in your valuable Journal would be of material service, in procuring an important addition to the information that may be expected from other channels, I beg leave to enclose a copy of it for that purpose; and I hope that your friends and correspondents will take an early opportunity of transmitting, either to me, or to the Publisher of the Medical and Physical Journal, any information connected with the particulars alluded to in the paper, which they may consider to be material.

*5, Terrace, Palace Yard, Westminster,* I am, &c.

*April* 23, 1805. JOHN SINCLAIR.

---

### PROSPECTUS, &c.

A few extracts from this prospectus will give the public an insight into the author's views, and the plan of his work:

" It is observed," says he, " though all must die, yet that some some individuals live for a shorter and some for a longer period; that while some perish during infancy or youth, the existence of others is prolonged for many years.

"It is also remarked, that while some individuals enjoy the possession of their mental and corporal powers, almost unimpaired, during the whole period of their existence, others are occasionally, and in some cases perpetually, in a sickly state; the one of course possessing the advantages of *health*, and the others being the victims of *disease*.

"It has farther been noticed, that many persons, by means of various remedies, which experience has pointed out, have recovered from those disorders with which they have been afflicted, and from the personal accidents which they have unfortunately undergone, so as to regain their former health and strength.

"Hence arises the threefold division of the general subject of Life and Health; namely, into the means, 1, *Of prolonging life*, to which some authors have given the name of the Macrobiotic Art; 2, *Of preserving health;* and, 3, *Of curing and alleviating disease.*

"Of the means by which persons are relieved from the various disorders and accidents to which they are liable, it is not the object of the intended volumes to treat. Such particulars comprehend the arts of medicine and of surgery. There a vast and extensive field opens itself; to the cultivation of which thousands of able men have directed their attention, and in many respects successfully, though these arts have not hitherto reached that degree of perfection which it is so desirable, for the comfort of human nature, they should attain.

"Of the other two objects, however, the means of prolonging life, and preserving a state of health (not as distinct propositions, but as being, *when properly viewed*, so intimately blended together, that they can hardly be separated) it is the intention of the author to treat. What inquiry can be of more real importance? If health be the greatest blessing of life, and if disease render man miserable, what can be more desirable, than to ascertain the means of preserving the advantages of the one, and of counteracting the ravages of the other? If by prolonging our existence also we can be of more service to mankind, from the superior knowledge which greater experience, and longer observation, must generally furnish, what can be more important, than to endeavour to preserve our health and strength, that we may be the better enabled to perform beneficial and useful actions? For the power of doing good is is the proper limit by which our wishes for existence ought to be bounded.

"When all these particulars are considered, it surely can require little apology from any person who has applied his mind to such subjects, for venturing to lay before the public his ideas concerning them.

"It may be proper, at the same time, briefly to explain how an individual, unconnected with the medical profession, has been led to pay such particular attention to the subjects of health and longevity, as to consider himself competent to the task of instructing others regarding such interesting particulars.

"Though naturally possessed of a sound constitution, untainted by any hereditary disease, yet, about six or seven years ago, the author had fallen into a weak and enervated state, and found himself unequal to the task of managing his own private concerns, of prosecuting useful inquiries, or of applying his mind to political pursuits, with his former energy and zeal.

"As age advanced, he found many of his contemporaries either getting into a declining state, or sinking into the grave, much sooner than he had expected. The causes of these events he naturally wished to ascertain, and to determine whether so premature a decay might not have been prevented.

"In carrying on his statistical inquiries (a branch of which, namely, the effects of climate, was necessarily connected with the points in question) it was a matter of astonishment to find how few of the human species, in proportion to the numbers born, attained any considerable extent of years, even in the healthiest countries.

"Above all, it was a matter of regret (and the longer one lives the more striking it becomes) that even while men do live, their existence is too often embittered by disease, and their life a burden both to themselves and to others.

"These circumstances, united, naturally directed his attention to the subjects of health and longevity.

"Having printed, both in English and French, a short treatise on health and longevity, accompanied with several questions, in order to obtain additional information, he has thence fortunately procured a variety of important communications, in manuscript, both from foreign and domestic correspondents, by which the intended work will be considerably enriched.

"But, in particular, he has to rely on the treasures of knowledge knowledge, regarding health and longevity, published by various authors who have already written on these subjects, and of whose works he has made a collection to the number of about two hundred distinct treatises or volumes, every one of which is, either directly or indirectly, connected with the proposed inquiry. From this immense mass he proposes to extract whatever seems to be peculiarly valuable.

"On subjects respecting which so many volumes have been written, it will not be easy to draw up a new work, the form of which will give universal satisfaction, as many will desire a more condensed, and many a more detailed explanation.

"To gratify the first, it is the author's intention to consolidate, into one volume, octavo, all the knowledge which he considers to be *essentially necessary* for the attainment of health and longevity. The generality of mankind will not have leisure to read, and many of them cannot afford to buy, a larger publication.

"But, for those who may be desirous of investigating the subject farther, he intends to print, in four volumes, octavo, distinct from the other work, but to be published at the same time, an account of the writings and opinions, 1, of ancient, 2, of foreign, and, 3, of British authors, who have discussed the subjects in question.

"The most natural division of the subject under consideration seems to be, to point out,

"1. The circumstances which necessarily tend to promote health and longevity, independent of individual attention.

"2. The rules which, if observed by an individual, have a tendency to preserve health and existence, even where these independent circumstances are wanting. And,

"3. The regulations by which the general health and safety of a great community are protected from the various injuries to which they are likely to be exposed.

"PART I.

"*Circumstances which necessarily tend to promote Health and Longevity.*

"It will hardly be disputed, that while individuals differ so much from each other, with regard to a variety of important particulars, as the climate in which they reside, the manner in which they are formed, &c. that there must necessarily be a material difference with respect to the duration of their lives. It is essential therefore, in the first first place, to ascertain what these particulars are. It seems to me, that they may be all comprehended under the following general heads:

1. Form and growth of the individual.
2. Natural constitution.
3. Disposition of mind.
4. Parentage.
5. Climate.
6. Education.
7. Rank in life.
8. Particular occupation.
9. Connubial connexion.
10. Sex.

" PART II.

" *Rules for preserving Health and promoting Longevity.*

" It is evident, that if men lived uniformly in a healthy climate, were possessed of strong and vigorous frames, were descended from healthy parents, were educated in a hardy and active manner, were possessed of excellent natural dispositions, were placed in respectable situations in life, were engaged only in healthy occupations, were happily connected in marriage, &c. &c. there would be little occasion for medical rules. But it is universally known, that some individuals enjoy only a part of those advantages, whilst others possess hardly any of them complete. Hence arises the necessity of attending to those *rules* which observation and experience have pointed out as being the most likely to counteract the disadvantages arising from so material a want, as of any of the natural advantages above enumerated. These rules relate to

1. Air.
2. Diet.
3. Digestion, and its effects.
4. Clothing.
5. Habitation.
6. Exercise of the mind.
7. Exercise of the body.
8. Sleep.
9. Amusements.
10. Habits.
11. Temper, or disposition.
12. Medicine.

" To which will be added, several rules of a *miscellaneous nature*, concerning the means of alleviating the effects of the various accidents to which persons are exposed; together with observations on the necessity of adhering to different rules, according to climate, peculiar occupations, &c.

" PART III.

" *Regulations for the Health of the Community.*

" The police of public health is a most important branch of the proposed inquiry; and the events which have re-

 cently cently happened in Spain, and at Gibraltar, have given it additional interest. It may be treated of under the following general heads:

1. Police of climate.
2. Police of physical education.
3. Police of diet.
4. Police of public amusements.
5. Police of habits and customs.
6. Police of public institutions.
7. Police for the health of sailors and soldiers. And
8. Police of medicine, and the means of promoting its improvement.

---

*Observations on the training of Pugilists, Wrestlers, Jockies, and others, who give themselves up to Athletic Exercises; with some Queries for discovering the principles thereof, and the process of training Running Horses, &c. with a view of ascertaining, whether the same can furnish any hints serviceable to the human Species.*

THERE are habits which seem to be natural to, and congenial with, the several periods of life. The child should merely suck, sleep, and vegetate. The boy should ramble wild and unconstrained, little oppressed with tasks or studies, and nourished with abundance of simple food. The youth should be temperate, sober, active. The old man quiet, sedate, self-indulgent; should have long sleep, delicate food, rich wines, and agreeable temperature; little labour, and a cheerful mind. Nature assigns us vigour, spirit, enterprise, and foresight, in the early part of life, to treasure up the needful indulgencies for age. Parents are careful of our first infancy; we ourselves ought to provide for our latter childhood.

Next to the free circulation of the blood, through all the body, terminating in the surface, that of the free transit of the blood through the lungs, is essential to health.

The oxydation or chemical change produced by air upon the blood, is essential to its vital properties. A free and powerful respiration is most essential to a fresh colour of the face, to lively spirits, and cheerful feelings, and to the healthy and vigorous actions of the body. "It is my breathing hour of the day," says Hamlet to Ostric. It is a princely thing to set apart hours for exercises; and there is little doubt, that if all those, who linger away their hours in luxurious and indolent relaxation, were to assign a regua regular portion of their time, to the hardy and manly exercises of walking, riding, fencing, &c. and would take *their breathing hour,* they would breath long and well.

These reflections naturally arise upon considering the almost incredible perfection, to which those, whose profession it is to train men to athletic exercises, have brought their respective arts. By certain processes, they improve the breath, the strength, and the courage of those they take in hand, so as to enable them to run thirty, or walk a hundred miles, in a given space of time; to excel in wrestling, or to challenge a professed boxer. Would it not then be a most important addition to the facts we already know concerning the means of improving strength, and ensuring long life, if authentic information could be procured from those districts where athletic exercises prevail, what are esteemed the best and surest processes for training men for foot-races, trials of strength in wrestling or boxing matches, or for raising the strength and courage of game-cocks, or improving the wind, strength, and speed, of running horses to their highest pitch.

I question whether the athletics of old used similar means; whether they were equally successful; whether there ever were, in any climate, age or country, more hardy or powerful frames than those of our English pugilists. In Cooke's voyage, we are told of the marked inferiority of the English sailors, in wrestling or boxing, to the naked sun-burnt heroes of the South Sea islands. But an English sailor, though full of spirit and vigour, is as clumsy as a clown, and could not even row against an inhabitant of the Sandwich Islands. An English bricklayer, blacksmith, or draymen, however, who liked the sport, and was practised in balancing and striking, might have challenged the whole of the tawny nation.

With a view of collecting such important information, I am very anxious that the following Queries should be proposed to those who profess the art of training pugilists, wrestlers, and runners of foot-races, by such intelligent men as have the opportunity of conversing with them.

1. By what criterions or tests, they judge of the muscular strength, or wind, or other qualities of those who seek to put themselves under training? What is the earliest, and what is the latest age they would attempt to train?

2. How they judge of the length of time that may be required for bringing a man into good plight, vigorous health, health, and free breathing? and what period of preparation is usually required for running a match?

3. What purges they use; and in what succession; and by what rules do they administer them; and how do they judge of their effects? Is the purging only preparatory, or is it regularly continued? Is it meant, by this process, to reduce the plethoric state of the system, (on the idea that there is too great a quantity of blood), or is it simply designed to put the bowels in the most favourable condition, for easy and good digestion? Is the reducing the actual size of the belly, necessary to more free and perfect breathing?*

4. Is the diet rich or simple; of animal food, or of vegetable; in great quantity, or sparing? Is it increased gradually, or diminished gradually? What meals have they in the day; and at what hours; one or more; frequent feeding, in small and fixed portions, or full and substantial meals? What kinds of flesh or meat is reckoned the best; whether beef, mutton, veal, pork, lamb, or fowl? Are any kinds of fish allowed? What quality of food is most conducive to strength? What quantity is necessary for maintaining the system in its most perfect state of vigour? Do they feed much in the intermediate days of the purges? Is abstinence required when they take their physic?

5. What kinds of liquors are reckoned best? Whether wine, ale, water, spirits, &c.? Whether given hot or cold; in what quantities; and when ought they to be given?

6. Are the very violent perspirations into which they throw their patients, designed to reduce the system, to extenuate the fat, to lessen that quantity of blood, the excess of which makes us giddy or short breathed; or is it merely designed to produce a new condition of the skin, more favourable to health, and muscular vigour; to produce a sharper appetite; a greater demand for food; and a quicker nourishment, or a greater nutrition from a more slender diet? Is the sweat at first produced by exercise, and only

* The effects of taking up a running horse from idleness and soft pasture, to hard food and regular exercise, is attended with this peculiar effect, that while the animal becomes lank, sleek and glossy, while he gets fire in his eye, and a new vigour in his limbs, and wind and speed, his belly, (swollen with coarse indigestible food, eaten in great profusion), is drawn into half its size. May we not then presume from this analogy, that the state of the belly has a remarkable effect upon the wind?

only continued by the person, when trained, being put between feather beds, and encouraged by drinks; or is it produced by force of sweating drugs, or violent heats, or by continued friction? At what hours are the perspirations brought on? How is the pupil treated when the sweat is over? What becomes of the skin of a fat man, when, by the process, he is reduced in size, and rendered lean? Does it hang loose, or is it tight? Has it any effect upon the bones?

7. What hours of exercise do they require of their pupils during the day? At what hours do they send them out in the morning? How long do they continue abroad? Are they loaded with clothes after the body is reduced, and becomes limber, and thin and muscular; or only while the sweating process continues? Are they fed before they go abroad, or when they return? What trials are made of their strength? When is a man known to be up to his full strength and breath in training? At what hours do they go to bed? What sleep are they allowed? What indispositions are they subject to during training? Are there any circumstances by which the process may be interrupted; or any circumstances, in consequence of hich, it must sometimes be abandoned.

8. What is the state of the health, after they give up training? Are they subject to any complaints; and what are they? How long does the acquired excess of strength continue?

9. It is most interesting to leain, on which part of this process, the purging, the sweating, the exercise, or the feeding, they most depend; and whether it procures a permanent increase of vigour, easily maintained by suitable diet and exercises, or only a temporary excitement, calculated for the particular occasion? Also, whether persons have ever thought of undergoing this process, not for the purpose of running matches, but to recover health; with what success this has been done, and whether it is to be recommended for gout, corpulency, asthma, nervous disorder, or other maladies, as likely to be of service?

These are questions, of the importance of which, those who are best able to answer, may not be fully aware. But nothing which so suddenly changes the powers, and the very form and character of the body, from gross to lean, from weakness to vigorous health, from a breathless and bloated carcase, to one active and untiring, can ever be unimportant, either to the art of physic in general, or to that branch of it, more immediately connected with inquiries regarding health and longevity.

The queries to be put regarding jockies, running-horses, or game-cocks, may be to the following effect:

### Jockies

1. What is the process used in training them, and reducing their weight?

2. What effect has it upon their health and strength?

3. What effect has it upon their mind, in regard to courage, quickness, &c.?

4. How long do these effects continue?

5. After being reduced, do they quickly get fat again, or do they continue long in the state to which they were brought?

6. Are jockies, accustomed to be thus treated, healthy and long lived?

### Running Horses.

1. What are the principal objects to be attended to in regard to running horses? Do their perfections depend upon parentage, and whether most upon the male or the female? Is it necessary that the mare should have gone her full time, to bring a perfect foal? Is the gradual growth of the foal essential? Is there a great difference, in regard to natural constitution, between horses of the same parentage? What kind of form is in general preferred? Do you prefer great or small bones? Which sex is preferable for speed, and which for strength?

2. What is the best age for beginning to train horses for the turf? Are they first put upon grass? What is the effect of soft meat? When should they be put on hard meat? What are the effects thereof? Is it necessary to purge them frequently? Have the purges any tendency to weaken them? What food is reckoned the most nourishing? How often are they fed? What drinks are given them, and how often? Whether hot or cold? Is it necessary to keep their skin perfectly clean, and how? Is it necessary to make them perspire much? What exercise is given them? How is the training completed?

3. After the training is completed, can the perfections thereby obtained be easily kept up? Does the process effect merely a temporary change, or does it last during life? Are running horses as long lived as others, or do they soon wear out?

GAME COCKS.

1. Does the superiority of game-cocks depend upon parentage? Which is of most importance, the male or the female? Is it of any consequence that the cock should arrive rather gradually at maturity? Is there a great difference in point of strength and constitution, in game-cocks of the same parentage? Do you prefer great or small bones?

2. When do you begin to feed the young cocks? What diet and drink do you give them, and what is the process by which they are brought to the greatest possible height of strength and spirit?

3. When the game-cocks are thus trained, how long do the effects thereof last? Are they temporary or permanent? Do game-cocks thus trained live shorter or longer than others of the same species?

4. What drugs are given to fighting-cocks immediately before the main begins? Is it not usual, by giving them saffron, (or some drug, which has the same effect with opium, as used among the Janisaries, or brandy among the French soldiery), to excite an unnatural and short-lived courage? What are the effects of such drugs? and how do they manage the feeding up to this point, so as to take advantage of this momentary excitement?

---

